# The magnitude of nephron number reduction mediates intrauterine growth-restriction-induced long term chronic renal disease in the rat. A comparative study in two experimental models

**DOI:** 10.1186/s12967-016-1086-3

**Published:** 2016-11-30

**Authors:** Farid Boubred, Laurent Daniel, Christophe Buffat, Michel Tsimaratos, Charles Oliver, Martine Lelièvre-Pégorier, Umberto Simeoni

**Affiliations:** 1 NORT, Aix-Marseille Université, INRA, INSERM, 13005 Marseille, France; 2 UPRES EA3281, Aix-Marseille Université, 13005 Marseille, France; 3 Laboratoire de Biologie médicale, AP-HM, Marseille, France; 4 Pédiatrie Multidisciplinaire-Hôpital de la Timone, Marseille, France; 5 INSERM U652, Institut Biomédical des Cordeliers, Paris, France; 6 DOHaD Laboratory, CHUV University Hospital and UNIL, Lausanne, Switzerland; 7 Department of Neonatology, Hôpital la Conception, AP-HM, 147 Boulevard Baille, 13385 Marseille Cedex, France

**Keywords:** IUGR, Low birth weight, Preterm infant, Nephron number, Prenatal glucocorticoids, Hypertension, Chronic kidney disease, Glomerular sclerosis

## Abstract

**Background:**

Intrauterine growth restriction (IUGR) is a risk factor for hypertension (HT) and chronic renal disease (CRD). A reduction in the nephron number is proposed to be the underlying mechanism; however, the mechanism is debated. The aim of this study was to demonstrate that IUGR-induced HT and CRD are linked to the magnitude of nephron number reduction, independently on its cause.

**Methods:**

Systolic blood pressure (SBP), glomerular filtration rate (GFR), proteinuria, nephron number, and glomerular sclerosis were compared between IUGR offspring prenatally exposed to a maternal low-protein diet (9% casein; LPD offspring) or maternal administration of betamethasone (from E17 to E19; BET offspring) and offspring with a normal birth weight (NBW offspring).

**Results:**

Both prenatal interventions led to IUGR and a similar reduction in birth weight. In comparison to NBW offspring, BET offspring had a severe nephron deficit (−50% in males and −40% in females, p < 0.01), an impaired GFR (−33%, p < 0.05), and HT (SBP+ 17 mmHg, p < 0.05). Glomerular sclerosis was more than twofold higher in BET offspring than in NBW offspring (p < 0.05). Long-term SBP, GFR, and glomerular sclerosis were unchanged in LPD offspring while the nephron number was moderately reduced only in males (−28% vs. NBW offspring, p < 0.05).

**Conclusion:**

In this study, the magnitude of nephron number reduction influences long term renal disease in IUGR offspring: a moderate nephron number is an insufficient factor. Extremely long-term follow-up of adults prenatally exposed to glucocorticoids are required.

## Background

Epidemiological evidence shows that exposure to an adverse foetal environment, manifested in part by intrauterine growth restriction (IUGR), increases the risk of cardiovascular disease (CVD) and chronic renal disease (CRD) in adulthood [1–3]. Prevalence of chronic renal disease, defined as reduced glomerular filtration rate (GFR), proteinuria or both is increasing in several countries [4]. Such an association has been reproduced in animals from various species but not in all experimental studies which suggest that factors others than IUGR may be involved [5–8]. In rats, a maternal gestational low-protein diet (LPD) and prenatal administration of synthetics glucocorticoids, namely betamethasone and dexamethasone, have been widely used to reproduce conditions of human IUGR [2, 9]. Both models share at least a common pathophysiological mechanism which is fetal overexposure to glucocorticoids. As demonstrated in human IUGR, maternal LPD is associated with fetal glucocorticoids overexposure due to down-regulation of placental type 2 11-β hydroxysteroid dehydrogenase (11β-HSD_2_) which inhibits transplacental transfer of maternal corticosterone [4]. Reduced transplacental amino-acid transfer has been shown in human IUGR as well [10, 11]. However most of the experimental studies included young adult offspring and long term patterns of blood pressure, renal functions and structure have been little investigated.

IUGR-induced hypertension and CRD are postulated to result from a nephron deficit [2, 12, 13]. In this case, according to the scheme of Brenner et al., single-nephron glomerular hyperfiltration (SNGHF) and glomerular hypertension occur to meet the excretory demands [14]. Over a long period, a vicious cycle occurs, leading to glomerular and tubular injury with proteinuria, glomerular sclerosis, impaired renal functions, and systemic hypertension. However, recent findings question this pathophysiological mechanism and suggest that a nephron deficit could be insufficient to induce systemic hypertension and CRD [15–19].

Using two different IUGR models (a maternal LPD and gestational administration of betamethasone (BET)), which led to a similar reduction in birth weight, we aimed to show how the size of the reduction in nephron endowment influenced renal functions and structure in ageing offspring.

## Methods

### Animals and ethical approval

All animal procedures were approved by the Institutional Animal Care and Use Committee of Aix-Marseille University and in accordance with the European Communities Council Directive (2010/63/EU). Male and female virgin Sprague–Dawley rats, with an initial weight of 225–250 g, were used (purchased from Charles Rivers, l’Abresle, France). After 2 weeks of acclimatization, males and females were mated overnight. The day on which sperm were seen in the vaginal smear was designated as day 1 of pregnancy. Rats were housed in a room with a 12-h light/dark cycle and maintained at a controlled temperature of 22 °C. They had free access to food and water. Pregnant rats were randomly assigned to the control group (normal gestation, n = 6) or one of two IUGR groups, namely, a maternal LPD (n = 6) and maternal administration of BET (n = 6). The maternal LPD contained 9% casein (vs. 18% in control diet) and was fed ad libitum during gestation. BET (0.25 mg/kg body weight) was administered intramuscularly at E17, E18 and E19. BET-administered pregnant rats were fed a normal diet ad libitum. After delivery, all dams were fed the same standard diet (18% casein). The weights of all pups were recorded within 6 h after delivery. The size of the litter was reduced to 10 pups, with the largest and the smallest pups removed, to ensure adequate and uniform nutrient supply to the pups. They were cared for by their mother until weaning on day 21, after which they were housed in groups of four. They had free access to water and the same standard laboratory rat chow. Offspring were weighed monthly until the end of experiments (22 months). For subsequent experiments, 2–3 male and female offspring were randomly selected from each litter.

### Systolic blood pressure (SBP) measurement

SBP was determined non-invasively using tail cuff plethysmography (Letica 5000, Bioseb, France) and thermostatically warmed restrainers designed for rodents. This method has been extensively validated in rodents and refined to reduce possible stress-related effects. Each animal was acclimatized to this procedure over 5 successive days (10 min per day). Thereafter, measurements were performed by a single operator. The mean of 4–6 measurements was recorded for each animal. SBP was measured at postnatal month 1, 4, 8, 12, 15 and 22. Unfortunately BP measurements in 22-month old offspring were inadequate and unusable due to unfit size of the tail cuff; also we decided not to consider the data.

### Determination of renal function

Endogenous creatinine clearance (ClCr) and the daily urinary protein excretion rate (UprV) were determined in 8-, 12-, and 15-month-old offspring as ClCr = UCr V/Pcr, where Ucr and Pcr designate the urinary and plasma creatinine concentrations, respectively, and V is the urinary output. Animals were housed individually for 48 h in metabolic cages. Urine excreted over 24 h and blood samples from the lateral tail vein were collected under brief general anaesthesia (isoflurane Belamont). Blood samples were then transferred to heparinized tubes and centrifuged at 3000 rev/min for 15 min at 4 °C. In 22 month-old animals urinary protein/creatinine ratio were assessed after anaesthesia. Plasma creatinine, urinary creatinine, and protein concentrations were measured using a standard autoanalyzer (Synchron LX 20 autoanalyzer, Beckman Coulter). The plasma creatinine concentration was determined by the method of Jaffé.

### Parameters of the renal structure

Nephron number and renal structure were evaluated in 1 day-old and in 22-month-old offspring from the three experimental groups. Animals were anaesthetised by intraperitoneal administration of pentobarbital sodium (60 mg/kg) with isoflurane inhalation. The left kidney was rapidly harvested, weighed, and decapsulated for glomerular counting, while the right kidney was removed, weighed, and kept for histology. In pups, mature and immature (S shape) nephrons were counted. Because gender was difficult to determine in 1 day-old pups and in order to avoid bias we decided not to express nephron number according to gender at that age. The number of glomeruli per kidney was determined using the dissection-maceration acid method as described previously [20]. Briefly, whole kidneys were incubated in 50% hydrochloric acid at 37 °C for time varying according the kidney weight (30–45 min). Kidneys were rinsed with tap water and stored overnight at 4 °C in a gauged flask. Following mechanical dissociation, tubules and glomeruli were suspended in water. Three 0.5-ml aliquots were taken and placed in a hemocytometer-like chamber. Glomeruli were counted under a microscope by three investigators who were unaware of the specimen’s origin. The three results were then averaged and the mean value was used to determine the total number of glomeruli in the sample and therefore the kidney.

Renal histology and corresponding parameters were analysed by a single investigator (L.D.) with no prior knowledge of the group to which the rats belonged. One-half of the right kidney was fixed in 4% buffered formaldehyde. The kidneys were then dehydrated using a graded alcohol series and embedded in paraffin. Transverse sections through the central portion of each kidney and 4-µm thick sections stained with haematoxylin and eosin were obtained for light microscopic examination. In each single section of the kidney, all glomeruli (i.e., superficial and juxtamedullary) sectioned through the hilum were counted. More than 80 glomerular cross-sections were analysed per group. The profile of a glomerulus was captured and the perimeter of the Bowman’s capsule was traced using a tablet cursor. The cross-sectional tuft area was calculated for each glomerulus with a visible vascular pole using image analysis (SAMBA 2005, Alcatel, TTITN Answare, France). The glomerular volume was then calculated assuming the glomerulus was spherical by applying the following mathematical equation: G_V_ = β/*k* × (G_A_)^3/2^, where β is the shape coefficient for a sphere (1.38), and *k* is the size distribution coefficient (1.1) [20]. Glomerular sclerosis was evaluated using Sirius red staining to visualise fibrillar collagen. Sirius red-stained fibrillar collagen as a percentage of the total glomerular surface area was thus determined. A single examiner (L.D.) performed this quantitative analysis using the same colorimetric and light thresholds (NCSS 2004 software, Kaysville, Utah, USA). A colour threshold was applied to identify the red-stained structures. The results were reported as the mean ratio of Sirius red-stained areas to total glomerular capillary areas.

### Statistical analysis

All data are presented as mean ± SEM and were evaluated for statistical significance using Statview version 5.0 software (Abacus Concepts Inc, Berkeley, USA). Normal distribution was verified using Kolmogorov–Smirnov test. Data for BP, GFR and proteinuria were analysed using 2 way ANOVA testing groups, postnatal age and groups × postnatal age interaction with Bonferoni post hoc analysis when appropriate. Body weight, renal structure parameters were compared between groups using one-way ANOVA with Student–Newman–Keuls comparison test for post hoc analysis. Male and female offspring were analysed separately. The interaction between group and sex was firstly evaluated and showed a sex effect for body weight, BP, parameter of renal functions and structure. A p value <0.05 was considered statistically significant.

## Results

### Birth weight and postnatal growth

Offspring prenatally exposed to maternal administration of BET (BET offspring) or a maternal LPD (LPD offspring) appeared healthy, although their birth weights were lower than those of control pups [normal birth weight (NBW) offspring] (6.47 ± 0.2, 5.08 ± 0.1, and 5.26 ± 0.1 g for NBW, LPD, and BET offspring, respectively; p < 0.001). The birth weights of LPD and BET offspring did not differ. The litter size and gender ratio were unaffected for both LPD and BET offspring (13 ± 2.2, 12 ± 2.5, and 14 ± 3 pups per litter for NBW, LPD, and BET offspring, respectively).

At the end of the suckling period, BET offspring remained lighter than LPD and NBW offspring (57.3 ± 1.1, 49.4 ± 0.9, and 41.8 ± 1.1 g for NBW, LPD, and BET offspring, respectively; group effect; p < 0.01; gender effect, p < 0.001; combined effect, p < 0.001). At the end of the study, when rats reached adulthood, the mean body weight of males NBW (703 ± 173 g) LPD (632 ± 151 g) and BET (617 ± 181 g) and females NBW (504 ± 141 g), LPD (484 ± 120 g), and BET (472 ± 149 g) offspring did not differ (group effect, p = 0.12; gender effect, p < 0.01; combined effect, n.s).

### Systolic blood pressure (SBP)

**Fig. 1:**
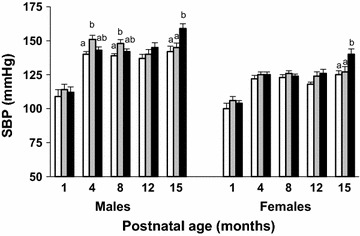
Long term hypertension in IUGR-BET offspring. Systolic blood pressure (SBP) of normal birth weight (NBW, *open bars*), maternal low-protein diet (LPD, *grey bars*), and BET (*black bars*) offspring from 1 to 15 months after birth, according to gender. Values are mean ± SEM; (n = 10–12/group). Significance differences (p < 0.05) among groups are indicated by different letters (e.g., *a* is different from *b* but not from *ab*). Group effect <0.001, postnatal age effect <0.001, and combined effects <0.001

During the experimental period changes in SBP differed among the three groups (Fig. 1). SBP increased over the experimental period for both male and female offspring (postnatal age effect p < 0.001). SBP was transiently higher in LPD male offspring than in NBW offspring at 4 and 8 months of age (p < 0.05) but was unaltered in female LPD offspring. By contrast, female and male BET offspring had significant HT when 15 month-old. SBP was 15 and 17 mmHg higher in female and male BET offspring, respectively, than in NBW and LPD offspring (p < 0.05).

### Renal function

**Fig. 2:**
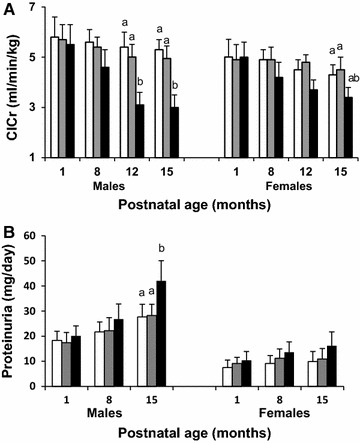
Renal functions in IUGR-BET offspring more affected than in IUGR-LPD offspring. Glomerular filtration rate [evaluated by clearance of creatinine (ClCr)] (**A**) and proteinuria (**B**) in normal birth weight (NBW, *open bars*), maternal low-protein diet (LPD, *grey bars*), and BET (*black bars*) offspring at 8, 12, and 15 months after birth, according to gender. Values are mean ± SEM (n = 10–12/group). Significance differences (p < 0.05) among groups are indicated by *different letters* (e.g., *a* is different from *b*). Group effect < 0.01, postnatal age effect <0.001, and combined effects <0.001

Clearance of creatinine (ClCr) and proteinuria (UprV) data are shown in Fig. 2. ClCr declined progressively with age in both males and females offspring (postnatal age effect p < 0.001). At 12 months of age, ClCr was 33% lower in BET male offspring than in NBW and LPD offspring (p < 0.05). Proteinuria was significantly increased in BET male offspring. ClCr and proteinuria were unaltered in LPD offspring. In 22 month-old offspring urinary protein/creatinine ratio (g/g) were significantly higher in BET offspring than other animals with 1.85 ± 0.1, 2.01 ± 0.08 and 3.06 ± 0.08 in male NBW, LPD and BET offspring respectively and 1.43 ± 0.1, 1.41 ± 0.06, 1.71 ± 0.1 in female NBW, LPD and BET offspring respectively (group effect p < 0.001; gender effect p < 0.001; combined effect p < 0.01).

### Renal structure

**Fig. 3:**
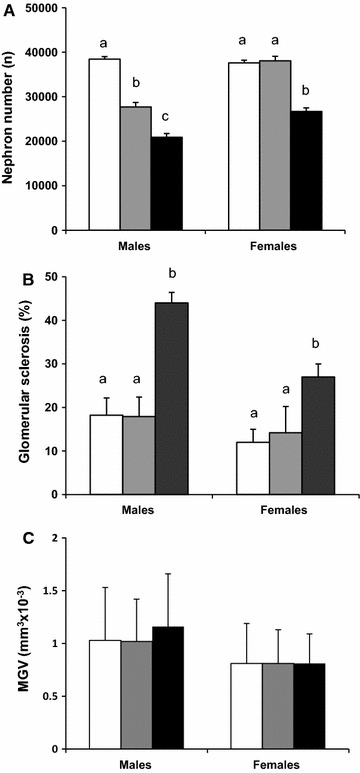
Renal structure in IUGR-BET offspring more affected than in IUGR-LPD offspring. Nephron number (**A**), glomerular sclerosis (**B**), and mean glomerular volume (MGV; **C**) in 22-month-old normal birth weight (NBW, *open bars*), maternal low-protein diet (LPD, *grey bars*), and BET (BET, *black bars*) offspring, according to gender (n = 8–12/group). Glomerular sclerosis is expressed as the mean ratio of Sirius red-stained areas to total glomerular capillary areas. Value are mean ± SEM; (n = 8–10). Significance differences (p < 0.05) among groups are indicated by *different letters* (e.g., *a* is different from *b* and *c*)

**Fig. 4:**
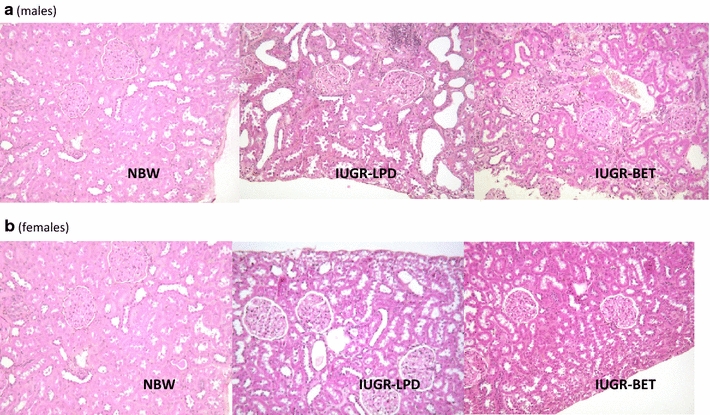
Histology of the kidney. Light microscopy examination of the cortical kidney of male (**a**) and female (**b**) normal birth weight (NBW), maternal low-protein diet (LPD), and betamethasone (BET) offspring. Kidneys of BET offspring exhibit glomerular sclerosis with interstitial inflammation. Magnification ×200

The parameters of renal structure in 22-month-old ageing offspring are summarized in Fig. 3. The relative kidney weight to body weight did not differ among the three groups, regardless of gender (2.06 ± 0.1, 2.01 ± 0.1, and 1.90 ± 0.2 for NBW, LPD, and BET offspring, respectively; groups and gender effects, n.s.). Nephron number was moderately reduced in male LPD offspring (−28% vs. male NBW offspring, p < 0.05) while it was unchanged in female LPD offspring. In comparison with male and female NBW offspring BET offspring showed 50 and 40% lower nephron number respectively (p < 0.001). Nephron number remained 22% (p < 0.05) and 45% (p < 0.01) lower in male and female BET offspring than LPD offspring. Such differences were already observed in 1 day-old pups when nephron number was more severely reduced in BET pups: 10488 ± 589, 6302 ± 223 and 5114 ± 145 in NBW, LPD and BET offspring respectively (p < 0.01). Renal morphology, including glomerular volume and glomerular sclerosis, was unchanged in 22-month-old male and female LPD offspring. But glomerular sclerosis was respectively 3.5-fold (p < 0.001) and twofold (p < 0.05) higher in male and female BET offspring than in NBW and LPD offspring (Fig. 3B and Fig. 4).

## Discussion

In this study, both prenatal interventions (a maternal gestational LPD and a short course of BET administration during a specific period of gestation) induced a similar level of IUGR, but altered blood pressure, renal functions, and renal structure differently. In comparison to LPD offspring, in which only males had a moderate nephron deficit and transient systemic hypertension, both male and female BET offspring had a severe nephron deficit, significant systemic hypertension, early renal dysfunction, and glomerular sclerosis. These findings suggest that a moderate reduction in nephron number alone is insufficient to induce these adult diseases. Although the pathological role of the kidney is still debated, a severe nephron deficit significantly enhances the risk of long term chronic renal disease (CRD) related to IUGR.

Despite a similar reduction in birth weight, nephron number was significantly lower in BET offspring than in LPD offspring. The protective factor of female gender commonly noted in LPD offspring was no longer observed in female BET offspring [21]. The last effect has not yet been reported. These findings confirm that short course of maternal gestational administration of glucocorticoids has specific effects on fetal nephrogenesis [22]. Betamethasone administered to pregnant dams crosses the placenta without being metabolized by the 11β-HSD_2_ and reaches the fetal kidney. The subsequent effects are highly significant when GCs are administered during the early stage of nephrogenesis [22, 23]. They are known to accelerate maturation at the expense of cell proliferation and to induce apoptosis. Expression of various genes have been found to be modified in the kidney from both IUGR models leading to apoptosis especially of nephron precursors, arrested branching morphogenesis and accelerated tubular maturation [24–27]. We also shown huge alterations in various genes especially involved in coagulation and inflammation pathways [28]. Inhibition of glucocorticoids production prevent nephron deficit in LPD rat offspring which strengthens the pathophysiological role of fetal overexposure to glucocorticoids [29]. Findings from our study and from other researchers, confirm that a short course of maternal administration of glucocorticoids at a specific time of gestation corresponding to early stage of nephrogenesis induces significant fetal nephron deficit.

A nephron deficit is proposed to be the mechanism linking IUGR and long-term systemic hypertension and CRD [10, 11, 30]. A nephron deficit is associated with pressure natriuresis re-setting and glomerular hemodynamic adaptations. According to the scheme proposed by Brenner et al. single-nephron glomerular hyperfiltration occurs to meet excretory demands [14]. Subsequent glomerular hypertension causes glomerular injury. Over a long time, a vicious cycle occurs, leading to proteinuria, glomerular sclerosis, impaired renal function, sodium retention and systemic hypertension. However, recent evidence questions this renal mechanism [13, 30, 31]. A nephron deficit is proposed to be a factor that confers vulnerability when other postnatal factors, such as postnatal overfeeding, rapid catch-up growth or high salt intake, accelerate or induce these diseases [6, 16, 18, 19]. We found that blood pressure, GFR and glomerular sclerosis were unaltered in LPD offspring with moderate nephron deficit suggesting that moderate nephron deficit alone is insufficient to induce these diseases. However, the severity of nephron deficit regardless of the reduction of birth weight plays a critical role. In contrast with LPD offspring, BET offspring born with similar birth weight reduction exhibited significant systemic hypertension, progressive renal dysfunctions and glomerular sclerosis. Lack of significant glomerular hypertrophy despite severe nephron number reduction may result from diffuse glomerular sclerosis associated with glomerular retraction (through ischaemia) [32]. All of these alterations were moderate in female BET offspring and seemed to be shifted in time. It is possible that another time point would reveal more significant changes. The main difference between the two groups was that BET offspring had more severe nephron deficit, already present at birth, which preceded the occurrence of systemic hypertension and CRD. Moreover BET offspring had a progressive postnatal growth that excluded the detrimental effect of a rapid catch-up growth. All these findings demonstrate that the magnitude of nephron number reduction highly influences long term IUGR-induced systemic hypertension and CRD.

In the current study, IUGR offspring had similar birth weight reduction but displayed different blood pressure and renal functions patterns. Systemic hypertension was transient in male LPD offspring only, as previously reported, and occurred later in BET offspring [2]. One can propose that these blood pressure patterns at young adulthood result from differences in stress response associated with blood pressure measurement. When using telemetry, the gold standard for measuring blood pressure in awake animals, blood pressure levels have been reported unchanged in 5-month-old LPD offspring but significantly lower in 8-month-old offspring exposed prenatally to dexamethasone in comparison with NBW rat offspring [33, 34]. In our study, we used the same plethysmography device in all animals and repeated blood pressure measurement at different ages might have reduced the stress of animals and limited biases. Further investigations are nevertheless required to evaluate differences in stress response between both IUGR models. In any case, all these findings justify very long term follow-up of blood pressure and renal functions/structure in IUGR offspring and highlight differences between IUGR models.

Our study has a few limitations. First we focused on the role of the kidney; however, other cardiovascular and metabolic systems known to drive the development of long term CVD may be differentially altered in both IUGR models [35, 36]. Impaired vascular structure, vascular endothelial dysfunction, increase sympathetic activity insulin resistance, hyper-leptinemia and hyperglycaemia have been reported in various IUGR models and in low birth weight infants. These functional and structural alterations promote systemic hypertension and in turn can affect renal functions and structure on the long term. But they are less likely to induce early renal dysfunctions in young BET offspring. Second we used clearance of creatinine (ClCr) to measured GFR which enable follow-up of animals but might underestimate subtle renal changes in few animals. Third one can question on the role of the severity of nephron deficit on the occurrence of renal disease since nephron deficit observed in ageing animal might be due to nephron loss related to ageing process rather than reduced nephron endowment. However the fact that nephron deficit was already present at birth prior to the development of systemic hypertension and CRD strengthens the pathophysiological role of severe reduced nephron endowment. Finally two different IUGR models were used to investigate the pathophysiological role of the kidney. It is noteworthy that varying the intensity of the prenatal insult (i.e., the amount of protein in the maternal gestational LPD or the dose of BET administered), may severely affect fetal growth and long term cardiovascular and renal functions and structure. The strength of our study was that IUGR-induced systemic hypertension and CRD were shown to be consequences of the severity of the nephron deficit, rather than the intensity of IUGR, because birth weight was similar in the two IUGR groups.

## Clinical implications

Extrapolation from this study to human IUGR should be made with caution since current findings were observed in specific experimental models. However some evidence argues for the potential relevance of the IUGR models for human IUGR. In human IUGR is a consequence of maternal undernutrition as observed in the developing world or of placenta insufficiency and preeclampsia mainly observed in industrialized world [37]. Both animal and human subjects IUGR share striking similarities such as impaired maternal–fetal amino-acid transfer and reduced placental 11β-HSD_2_ [9–11]. Moreover, our findings highlight the need for extremely long term follow-up of infants and adults exposed prenatally to glucocorticoids. Long term consequences are little known [38–40]. In pregnant women, glucocorticoids are prescribed for many reasons including acceleration of maturation of fetal lung in cases of threatened preterm labour, treatment of connective tissue disorders, and women at risk of bearing fetus with congenital adrenal hyperplasia. In this last case, dexamethasone is administered from the first trimester to suppress fetal androgen overproduction. A short course of glucocorticoids prescribed for women at risk of preterm birth, reduces neonatal mortality and morbidity but may have long term consequences [41]. Maternal glucocorticoids administration, especially at high cumulative doses, can affect fetal growth [39]. A trend in reduction of GFR and markers of insulin resistance have been reported in young adult born preterm and exposed prenatally to a short course of glucocorticoids as observed in experimental studies [39, 40]. These changes make them at high risk of cardiovascular and renal diseases.

## Conclusion

In the current study, we demonstrated that a maternal gestational LPD and administration of BET both led to a similar level of IUGR. However, maternal gestational administration of BET during a specific period of gestation more severely affected nephron number and induced systemic hypertension, early renal dysfunctions, and glomerular sclerosis in ageing offspring. Renal functions and structure were unaltered in ageing LPD offspring, who had a moderate nephron deficit. These findings suggest that a moderate reduction in nephron number alone is not sufficient to induce cardiovascular and renal diseases. Although the pathological role of a nephron deficit is still debated, IUGR-induced long term systemic hypertension and CRD depends in large part on the magnitude of nephron number reduction. Our findings highlight the importance of developing early markers of nephron number dosing and the need for extremely long-term follow-up of adults exposed prenatally (or postnatally for preterm infants) to glucocorticoids, even if the current data are reassuring.

## Abbreviations

BET: betamethasone
ClCr: clearance of creatinine
CRD: chronic renal disease
IUGR: intrauterine growth restriction
LPD: maternal low-protein diet
SBP: systolic blood pressure
UprV: daily urinary protein excretion rate
11β-HSD_2_: type 2 11-β hydroxysteroid dehydrogenase
